# Limitation in Controlling the Morphology of Mammalian Vero Cells Induced by Cell Division on Asymmetric Tungsten-Silicon Oxide Nanocomposite

**DOI:** 10.3390/ma13020335

**Published:** 2020-01-11

**Authors:** Hassan I. Moussa, Wing Y. Chan, Megan Logan, Marc G. Aucoin, Ting Y. Tsui

**Affiliations:** 1 Department of Chemical Engineering, University of Waterloo, Waterloo, ON N2L 3G1, Canada; h2moussa@uwaterloo.ca (H.I.M.); w33chan@edu.uwaterloo.ca (W.Y.C.); m3logan@uwaterloo.ca (M.L.); marc.aucoin@uwaterloo.ca (M.G.A.); 2 Waterloo Institute for Nanotechnology, University of Waterloo, Waterloo, ON N2L 3G1, Canada

**Keywords:** mammalian cells, morphology, adhesion, tungsten, silicon oxide, nanoscale

## Abstract

Engineered nanomaterials are often used in tissue engineering applications to influence and manipulate the behavior of cells. Recently, a number of tungsten-silicon oxide nanocomposite devices containing equal width (symmetric) tungsten and silicon oxide parallel line comb structures were developed and used by our group. The devices induced over 90% of seeded cells (Vero) to align within ±20° of the axes of 10 µm wide tungsten lines. Furthermore, a mathematical model was successfully developed to predict this alignment behavior and forecast the minimum width of isolated tungsten lines required to induce such behavior. However, the mechanism by which the widths of the symmetrical tungsten and silicon oxide lines induce the alignment behavior is still unknown. Furthermore, the model was never tested on more complex asymmetrical structures. Herewith, experiments were conducted with mammalian cells on complex asymmetrical structures with unequal tungsten and silicon oxide line widths. Results showed that the model could be extended to more complex pattern structures. In addition, cell morphology on the patterned structures reset during cell division because of mitotic rounding, which reduced the population of cells that elongated and aligned on the tungsten lines. Ultimately, we concluded that it was impossible to achieve a 100% alignment with cells having unsynchronized cell cycles because cell rounding during mitosis took precedence over cell alignment; in other words, internal chemical cues had a stronger role in cell morphology than external cues.

## 1. Introduction

Nanocomposites have a wide range of applications in electronics [1], mechanical structures [2,3,4], sensors [5], and bioengineering [6,7]. The ability to manipulate the behavior of biological cells by means of engineered nano-biomaterials is possible and effective [8,9,10,11,12,13,14,15]. Jahed et al. [14] demonstrated that the shape of 3T3 Swiss albino fibroblasts can be influenced by patterns of microscale silicon pillars. In addition to the overall shape of the cell, vertical pillars can also alter cell nuclear geometry in prostatic cancer cells (PC3) [11]. Moussa et al. [10,15] showed that both mammalian kidney epithelial (Vero) and human dermal fibroblast cells (GM5565) elongate and align on smooth-flat silicon oxide surfaces embedded with parallel tungsten (W) lines. In addition, similar cell alignment morphology was observed in adherent Japanese quail fibrosarcoma cell line cells (QT-35) on these devices as shown in Figure S1. This type of cell behavior has also been observed on textured tantalum [8], tantalum/silicon oxide composite [9], silicon [13], and hydrogel [12] topographic surfaces.

In most, if not all cases, however, there is a small fraction of cells that do not align with the line axes as expected; thus providing evidence that manipulating cell’s morphology does not prevent underlying life-cycle events. During cell division, a cell adhered on a smooth substrate contracts and becomes more spherical [16,17,18,19]. This characteristic mitotic rounding is accomplished by the rearrangement of actin micro-filament, which alters the geometry of adherent cells. Generally, cells maintain a near circular geometry during cell division. Lancaster et al. [16], Dix et al. [17], and Lancaster and Baum [18] all showed that mitotic rounding and reshaping of cells are critical features to ensure that mitotic assemblies are stable and properly formed (e.g., actin filaments, bipolar spindles, and microtubules). Lancaster [16] studied cell division in HeLa cells. The results showed that if cell height is constrained by external compressive stress or physical confinement during mitosis, the rate of mitosis related defects such as multi-polarity and mortality increases. Dix et al. [17] revealed that important components linked to focal adhesion in human RPE1-hTERT cells, such as zyxin, are lost during the cell division process, while active β1-integrin receptors remain at the cell–substrate interface. The latter is important as Petridou and Skourides [19] showed that cell β1-integrin activation influences the spindle capture site and affects cell division orientation. Failed activation of this receptor at the cell cortex or its symmetric distribution can ultimately lead to misorientation of the spindles. Therefore, cellular rearrangement is a natural phenomenon that cell manipulation devices should not restrict.

In earlier work, Moussa et al. [10] developed a mathematical model that predicts the alignment of Vero cells on simple smooth surfaces consisting of alternating tungsten and silicon oxide parallel lines. The model is based on maximizing the total surface area of the cell that can contact a preferred surface (in that case, a preference for tungsten). Given the simplicity of the model, it should be expected that if the alignment and patterning of cells is a maximization function, surface patterns can be altered and cell behavior can be predicted. Therefore, the first objective of this work is to investigate the alignment behavior of cells on smooth surfaces with asymmetrical features, i.e., patterns of alternating silicon oxide and metal parallel lines having different widths. As a second objective, the mathematical model developed by Moussa et al. is validated for more complex structures. This is important given that the majority of cell manipulation devices use complex patterns [20,21].

Finally, it is our hypothesis that even under the most favorable experimental conditions, the reshaping of cells during mitosis is one of the reasons a small population of cells remains misaligned. This is thought to be the direct result of cells maintaining a near spherical shape during the cell division process. Hence, it is statistically unlikely, at any time during the experiment, to have all cells aligned in the desired direction, if the cells are incubated for longer than the cell doubling time and have not had their cell cycle synchronized. Therefore, the third objective of this work is to demonstrate that mitotic rounding can be a limiting factor for cell manipulation on engineered surfaces. Vero cells are from an industrially relevant adherent cell line that require surfaces to grow. They are commonly used in virus infection [22] studies and were chosen for their ease of culture. It is also believed that, although they are transfectable [23], the cell line, and its transfectability, can be enhanced by manipulating the cells’ morphologies.

## 2. Materials and Methods

### 2.1. Asymmetric Parallel Line Comb Structure Surfaces

The chemical-mechanical polished (CMP) tungsten-silicon oxide nanocomposite was prepared with the same fabrication techniques described in our previous work [10] and supplied by Versum Materials, LLC (Tempe, AZ, USA). The process to fabricate the parallel line patterned structures is schematically illustrated in Figure S2. Briefly, a thin layer of silicon oxide was deposited on bare silicon substrate. Patterns were transferred on the silicon oxide by using the lithography and etch methods. This was followed by a tungsten deposition process to fill the trenches. Excess tungsten was removed by using the chemical-mechanical polishing (W-CMP) technique. Surface specimens contained alternating asymmetrical tungsten and silicon oxide lines with widths in the range of 1 and 100 μm. Details of the specimen structure geometries are summarized in Table 1. Typical top-down scanning electron micrographs (SEM) of the specimens are shown in Figure S3. These line dimensions were selected based on Moussa et al.’s [10] cell adhesion results on symmetric parallel line patterns. They showed that preferential cell alignment/elongation on tungsten lines are noticeable when the line widths and spacing are larger than 1 μm, but smaller than 100 μm. The increment of the line width used was in the range of ~2× to ~5×. This generated a well distributed dataset that covered a large range of line widths.

### 2.2. Cell Culture, Fixation, and Chemical Staining

Mammalian kidney epithelial (Vero) cells were acquired from the American Type Culture Collection (ATCC, Manassas, VA, USA). The detailed cell culture, fixation, and staining protocols were described elsewhere [10]. The initial cell concentration in the media was ~1 × 10^5^ cells/mL. Briefly, after 24 h of incubation on the tungsten-silicon oxide nanocomposite, adherent cells were fixed with 4% methanol-free formaldehyde (Sigma-Aldrich, Oakville, ON, Canada) and then dehydrated using a protocol that was described previously [10]. These specimens were used for producing SEM micrographs. However, specimens intended for fluorescence confocal imaging were fixed as stated above, permeabilized with 0.1% Triton-X 100 (Sigma-Aldrich, Oakville, ON, Canada), and then stained with deep red CytoPainter F-Actin stain (ab112127 Abcam, Cambridge, MA, USA) solution and 4′,6-diamidino-2-phenylindole (DAPI, Life Technologies, Waltham, MA, USA). Specimens were stored in a 4 °C refrigerator prior to fluorescence confocal imaging.

### 2.3. Scanning Electron and Fluorescence Confocal Microscopy

A field-emission scanning electron microscope (Zeiss 1550, Carl Zeiss AG, Oberkochen, Germany) was used to study the cell morphology. The distributions of microfilaments and deoxyribonucleic acid (DNA) were characterized using a fluorescence confocal microscope (Leica TCS SP5, Wetzlar, Germany) at the University of Guelph, Guelph, Ontario.

## 3. Results

### 3.1. Morphology of Adherent Cells

**Figure 1:**
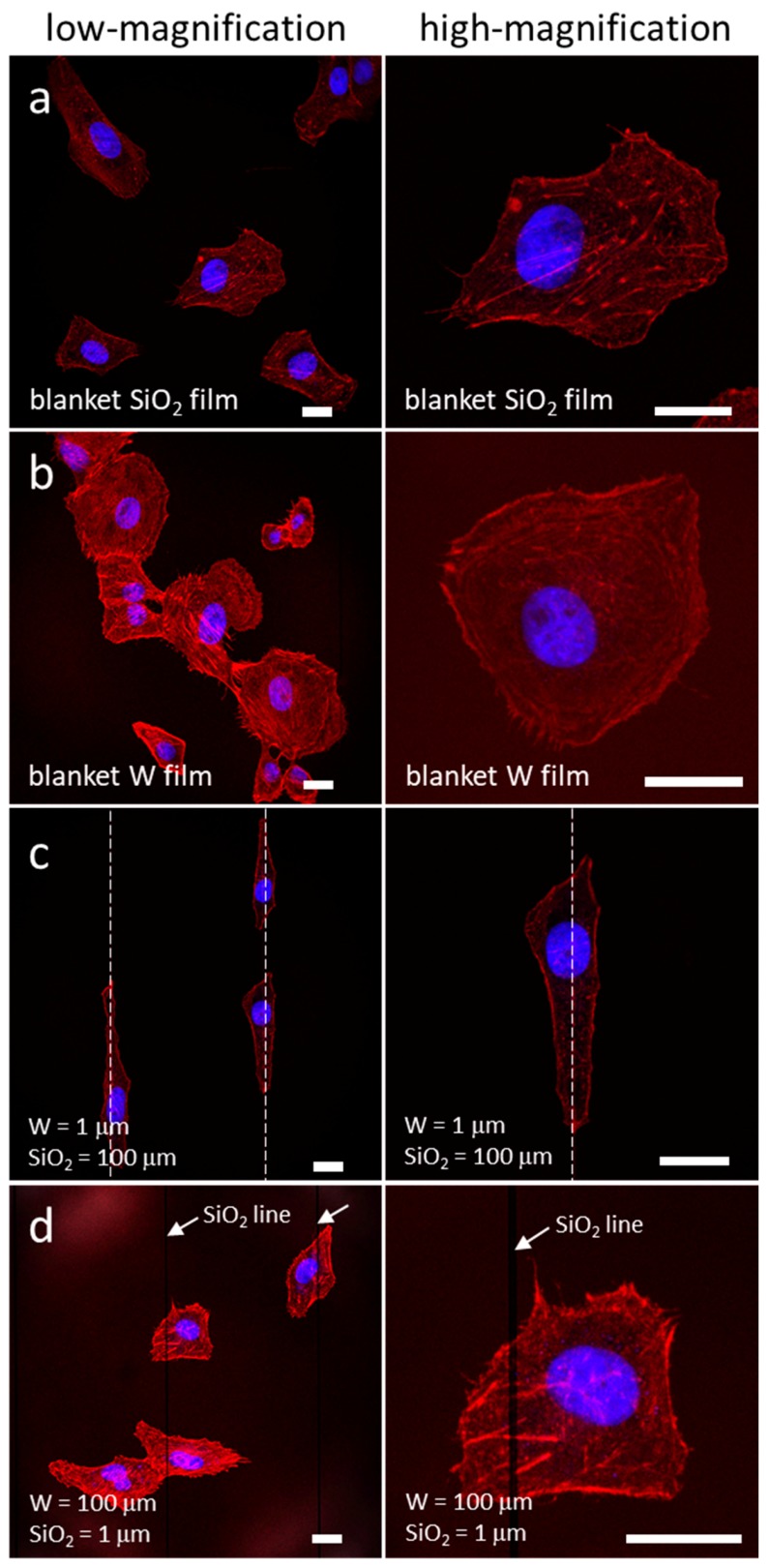
Low and high magnification fluorescence confocal micrographs of adherent cells on blanket (**a**) SiO_2_ and (**b**) W films. Aligned cells on the comb structures with a W line width of 1 μm and SiO_2_ of 100 μm are shown in (**c**). (**d**) shows irregularly shaped cells on the comb structure with 1 μm wide SiO_2_ lines separated with 100 μm of W. Cell concentration is ~1 × 10^5^ cells/mL incubated for 24 h. Scale bars represent 20 μm.

Typical low and high magnification fluorescence confocal micrographs of adherent cells on blanket silicon oxide (SiO_2_, field oxide) and tungsten (W) surfaces (field tungsten) are shown in Figure 1a,b. Adherent cells were randomly distributed and exhibited no preferential orientation.

In contrast, cells that adhered to the asymmetric line patterns, where tungsten lines had a width of 1 μm and silicon oxide lines had a width of 100 μm, were elongated and aligned with the 1 μm tungsten line (Figure 1c). The locations of the tungsten lines are highlighted with white dashed lines. When the line widths were inverted (tungsten lines had a width of 100 μm and silicon oxide lines had a width of 1 μm), the cells no longer displayed a preferential orientation (Figure 1d).

**Figure 2:**
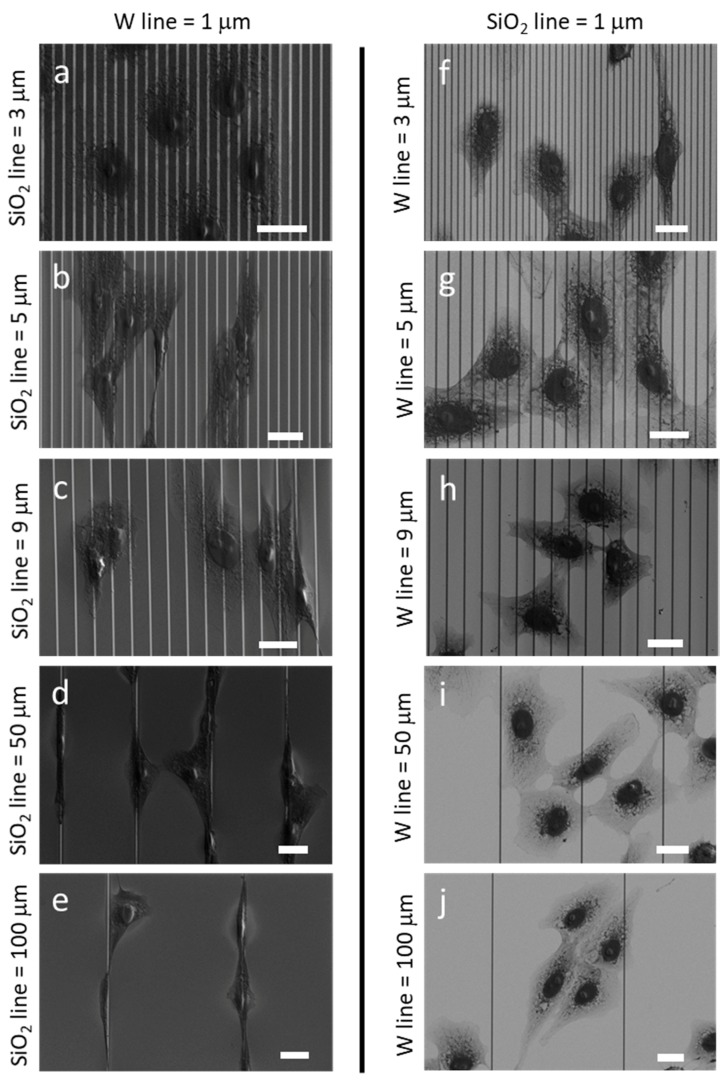
SEM micrograph of adherent cells on comb structures with different combinations of tungsten and silicon oxide line widths. (**a**–**e**) show substrates with a fixed tungsten line width of 1 μm and silicon oxide line widths of (**a**) 3 μm, (**b**) 5 μm, (**c**) 9 μm, (**d**) 50 μm, and (**e**) 100 μm. Additional substrates consist of fixed 1 μm silicon oxide lines and tungsten lines with widths of (**f**) 3 μm, (**g**) 5 μm, (**h**) 9 μm, (**i**) 50 μm, and (**j**) 100 μm. Scale bars represent 20 μm.

Scanning electron micrographs (SEM) of adherent cell morphology on other complex line patterns are shown in Figure 2. SEM images clearly differentiated between tungsten metal lines in light gray and silicon oxide lines in dark grey. The micrographs in the left column from (a) to (e) showed comb structures containing 1 μm fixed width tungsten lines with alternating silicon oxide lines with increasing widths of (a) 3 μm, (b) 5 μm, (c) 9 μm, (d) 50 μm, and (e) 100 μm. These images demonstrated a trend of increasingly aligned cells in the direction of the tungsten line axes as the width of the silicon oxide line increased. In contrast, cells on structures with the same line geometrical patterns, but with inverted material placement, showed a weak orientation preference, as seen in Figure 2f,j. These structures contained 1 μm wide silicon oxide lines and various tungsten line widths of (f) 3 μm, (g) 5 μm, (h) 9 μm, (i) 50 μm, and (j) 100 μm.

### 3.2. Quantitative Analyses of Cell Alignment

**Figure 3:**
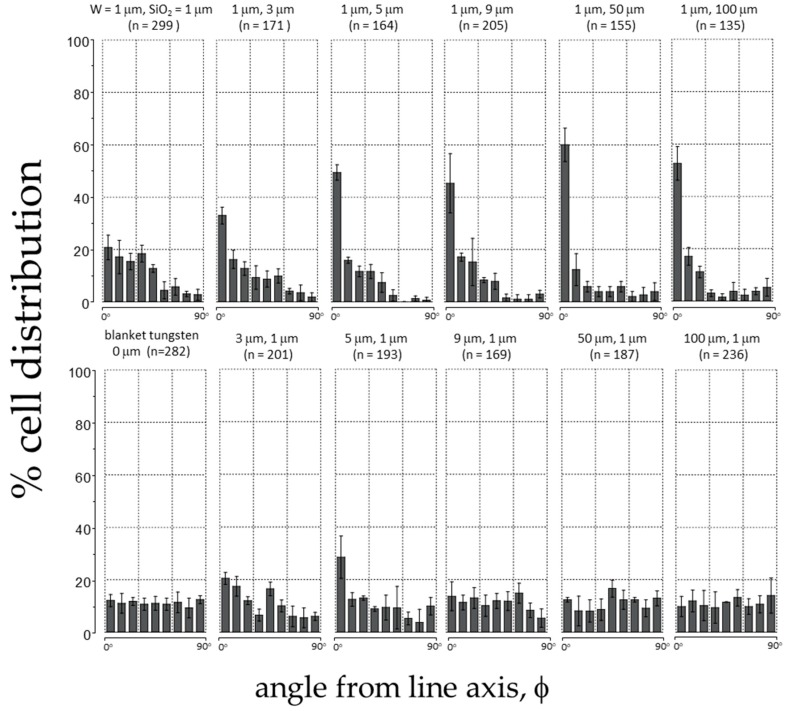
Percent cell distribution of cell orientation relative to the line axes (φ) after being incubated on substrates with various tungsten and silicon oxide line widths. Each bin corresponds to the cell population within a 10° angular range.

The angle (φ) between a cell nucleus’ long axis and the parallel line axes (illustrated in Figure S4) allowed the characterization of alignment behavior. For cells that were undergoing mitosis i.e., mitotic rounding, their orientation was characterized by the long axis of the entire cell. Cells were binned in 10° increments, which allowed for an alignment distribution as seen in Figure 3. Error bars correspond to one standard deviation from three independent groups of data. The number of cells measured in each comb structure (*n*) is included in the corresponding plot. Results from specimens with 1 μm fixed width W lines are shown in the top row of panels, while results from structures with 1 μm fixed width SiO_2_ lines in the bottom row. Results from the 1 μm × 1 μm comb structure and the blanket tungsten film are also included for comparison in the same Figure 3. The percentage of cells that were aligned within ± 10° of the W line increased as the distances between the 1 μm fixed width W lines increased (see top panels). The cell alignment performance reached a peak value of ~60% for W lines separated by alternating 50 μm wide SiO_2_ lines. The alignment performance of cells on structures with fixed 1 μm wide SiO_2_ lines was poor as shown in the bottom panels. Cells aligned the most (~30%) on comb structures with W lines of 5 μm wide.

### 3.3. Agreement with the Established Model

The mathematical model developed by Moussa et al. [10] was also tested using the same tungsten and silicon oxide alternating line comb structures as discussed in the experiment. The percentage of cell area that was in contact with W on various patterns was simulated. Two potential cell geometries were examined: irregularly and elongated shaped cells. Schematic drawings of these two cell geometries are illustrated in Figure S5. The percentage of a cell area on tungsten was calculated as:

(1) $$\%\;of\;cell\;contacting\;tungsten=\frac{cell\;area\;contacting\;tungsten}{total\;cell\;area}\;\left(100\right)$$

**Figure 4:**
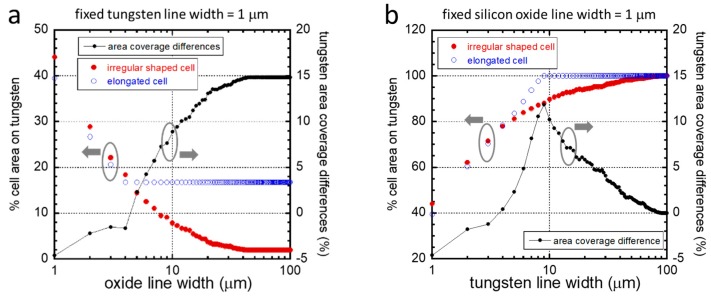
(**a**) Simulation result of the percent cell area in contact with W on parallel line comb structures. The tungsten line width was fixed at 1 μm in all models, while silicon oxide line widths varied in the range of 1 μm and 100 μm. (**b**) Simulation result for cells on patterns with the silicon oxide line width fixed at 1 μm in all models, while the W line width varied in the range of 1 μm and 100 μm. Tungsten area coverages for irregularly shaped (red close circles) and elongated (blue open circles) cells are plotted on the left axis. The nominal differences in the percent area coverage between the two cell geometries (black dotted line) are plotted on the right axis.

For different patterns, a percent cell area was calculated based on the geometry of our model cells (Figure S5), and the simulation results are plotted in Figure 4. The percentage tungsten area coverage differences (black dotted line), which is the difference between the elongated (blue circles) and irregularly shaped cells (red circles), is believed to be proportional to the driving force that induces the cell to elongate and align in the direction of the line axes. Figure 4a indicates that when the W lines were fixed at 1 μm, cells would have a greater tendency to elongate as the width of the silicon oxide lines increased; however, for SiO_2_ lines with a width larger than 50 μm, the driving force for alignment no longer increased, and the behavior should be stable. Figure 4b examines the inverted line pattern. With fixed 1 μm SiO_2_ lines, the driving force (black dotted line) increased to about 9 μm, after which there was no potential for alignment because the cell would presumably fit entirely on the W line. In fact, on wider tungsten lines, the driving force for cell alignment decreased. The entire population of cells on structures with tungsten line widths larger than 90 μm was expected to be arbitrarily oriented. This was the point where the driving force approached zero.

**Figure 5:**
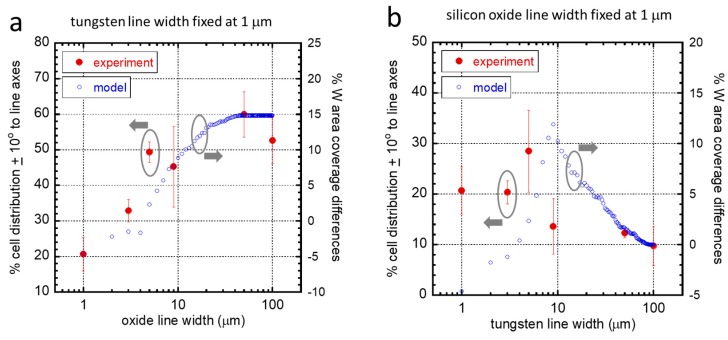
(**a**) Experimentally measured results of the percent distribution of cells aligned within 10 degrees of the comb structure line axes are plotted as a function of silicon oxide line widths on the left axis. The tungsten line width of these structures was fixed at 1 μm. Simulation results of the nominal tungsten coverage area differences between irregularly shaped and elongated cells are plotted on the right axis. (**b**) Comparison of experimental and model results for cells on patterns with the silicon oxide line width fixed at 1 μm and varying W line widths.

To validate these simulations, data from the mathematical model and from our experiment are plotted in Figure 5a,b. The experimentally measured cell population (red solid circles) that was aligned within ±10° of the lines axes is plotted on the left axes. The nominal difference of the modeled cell–tungsten area coverage between irregularly shaped and elongated cells is plotted on the right axes (blue open circles). For cells that adhered on structures with 1 μm fixed width W lines (Figure 5a), the dependency of both the cell distribution and the tungsten coverage on SiO_2_ line widths was remarkably similar. For the inverted patterns, again, experimental and simulated data showed similar important characteristic features, such as a peak in the cell alignment performance on structures with SiO_2_ line widths of 5–10 μm and reduced cell alignment with silicon oxide line widths larger than ~10 μm. These agreements suggested that the current selective adhesion model developed by Moussa et al. could predict the cell morphology on complex structures with asymmetric line widths.

#### Non-Aligned Cells

It is important to note that despite the ability to align most cells, all surfaces contained cells that did not align completely. In this work, under the best alignment condition, 40% of the population did not fall into the ±10° bin (Figure 3). Furthermore, the distribution of cells in the other bins was more or less uniform (an indication that there must be some underlying phenomenon interfering with the alignment process). Given that the cells had not been synchronized in their cell cycle, it is believed that this natural process, the process of cell division, was the major reason behind our observations. During mitosis, cells tend to round up [24,25], resulting in a considerable reduction in their total surface area [26], while temporarily losing their preferential adhesion and alignment behaviors induced by the substrate topographical features. Given that the doubling time of Vero cells is 24 h, the number of cells in various phases can be estimated [27]. For example, after 24 h of incubation, Quesney et al. [27] showed that ~14% of the cells were in the G2-M phases with a nearly circular shape geometry. With this in mind, we can expect ~14% cells to not align like the others. In our previous work, after 48 h of incubation on a 10 μm tungsten and silicon oxide parallel line comb structure, ~87 ± 1% of cells were aligned within ± 10° of the tungsten lines axes [10].

### 3.4. Influences of Cell Division on Cell Morphology and Alignment on Various Surfaces

#### 3.4.1. Uniform Tungsten Surfaces

**Figure 6:**
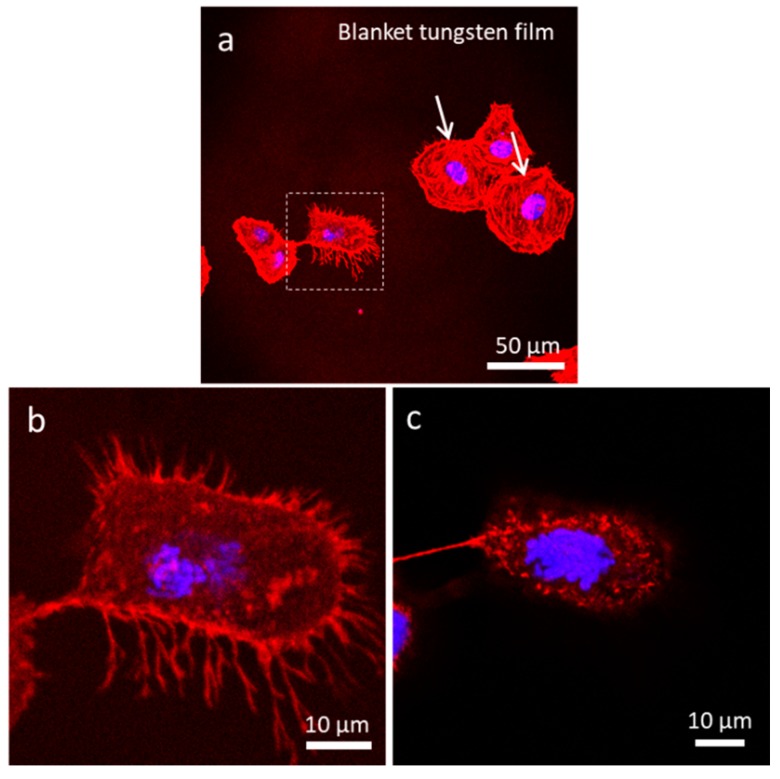
(**a**) Low magnification of a dividing cell possibly in the metaphase on blanket tungsten film. Images (**b**,**c**) show actin filaments protruding from the bottom half of the cell, while condensed DNA concentrated near the top of the cell (**c**).

To test the above hypothesis, cells were stained with two fluorescent dyes: CytoPainter F-Actin and DAPI, and imaged using confocal microscopy. The organization of actin filaments and DNA, which can be observed using these dyes, can reveal the life stage of the cell. As our control, cells were deposited on a uniform tungsten surface. Cells were randomly aligned, and the majority of the cells were spread out over the surface (Figure 6). A small fraction, however, displayed typical dividing cell behavior, where cells tended to contract laterally and increase in thickness, producing a near circular shape. An example of such behavior is shown in Figure 6a (dotted square box). A dividing cell appears to be in the prophase stage of mitosis. High magnification micrographs of this dividing cell showed actin filaments projecting outwards from the cell. Z-stack images of this cell are presented in Figure 6b,c and Figure S6, which cover focal planes near the surface all the way up to the top of the cell. While the actin filaments were concentrated towards the lower half of the cell, DNA was concentrated in the middle of the cell, bulging upwards like the yolk of a fried egg sunny side up. Detailed fluorescence z-stack images of this dividing cell are revealed in Supplementary Figure S6. Micrographs of another cell on blanket W film in the telophase stage of mitosis are provided in the Supplementary Data (Figure S7). Again, the irregular shape of the cell was attributed to cell division.

#### 3.4.2. Patterned Comb Structures (Alternating Silicon Oxide (1 μm) and Tungsten Lines (9 μm))

**Figure 7:**
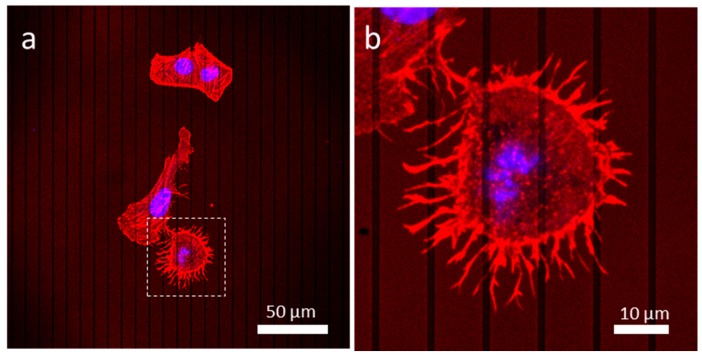
(**a**) Low magnification of a dividing cell possibly in the prophase on the structure containing alternating 9 μm tungsten lines and 1 μm silicon oxide lines. (**b**) High magnification image shows actin filaments protruding from the bottom half of the dividing cell.

On asymmetric comb structures with alternating silicon oxide (1 μm) and tungsten lines (9 μm), the behavior of the cells was very similar to that of cells on a uniform tungsten surface (Figure 7), where the majority of cells were not aligned with the line axes. Again, a cell, likely in prophase, highlighted with a dotted box, was more circular with its center protruding upwards just like the cell highlighted in Figure 6. Z-stack images of these cells are shown in Supplementary Figure S8. These micrographs revealed that the dividing cell was significantly thicker than the adjacent cell, which was in the interphase stage.

#### 3.4.3. Patterned Comb Structures (Alternating Silicon Oxide (5 μm) and Tungsten Lines (5 μm))

**Figure 8:**
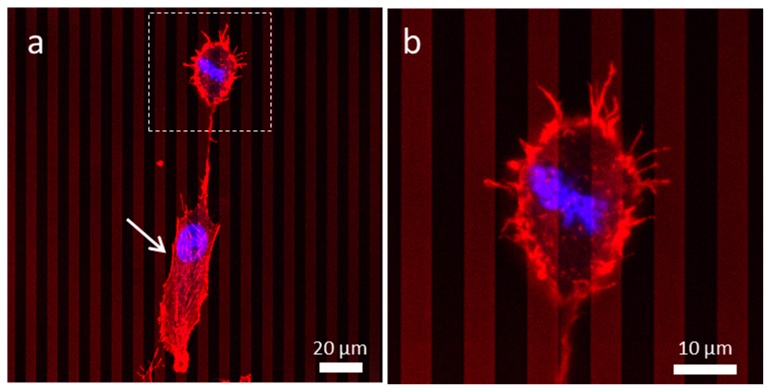
(**a**) Low magnification of a dividing cell possibly in the metaphase on the structure containing alternating tungsten and silicon oxide lines of an equal width of 5 μm. (**b**) High magnification image shows actin filaments protruding from the bottom half of the cell.

Figure 8a shows a cell in the interphase stage, labelled with an arrow, which is fully spread and aligned near the line axes. In contrast, a neighboring cell is in the metaphase stage with near circular geometry and is highlighted by a dotted box (see Figure 8a,b). The micrograph showed aligned condensed chromosomes at the center of the cell, and the metaphase plate was oriented at ~−53° from the line axes. Unlike the neighboring cell that was in the interphase stage, this dividing cell was not elongated extensively and did not show a tungsten dependent selective adhesion preference. The z-stack images shown in Supplementary Figure S9 also confirmed that the dividing cell was thicker than the adjacent cell in the interphase stage. Actin filaments protruded from this cell adhered to both silicon oxide and tungsten lines. This suggested that the preferential adhesion to tungsten may be reduced during mitosis, which led to a misalignment induced by the change in the morphology of cells throughout the time course of each division cycle.

#### 3.4.4. Patterned Comb Structures (Alternating Silicon Oxide (9 μm) and Tungsten Lines (1 μm))

**Figure 9:**
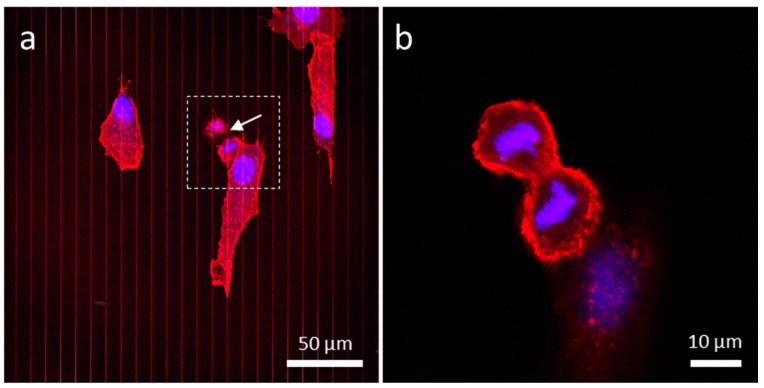
(**a**) A dividing cell possibly in telophase on the structure containing 1 μm tungsten and 9 μm silicon oxide lines. Other cells in the interphase stage are aligned with the line axes. (**b**) High magnification of the dividing cell highlighted in (**a**). It shows that the dividing cell is near spherical in shape and not aligned to the line axes.

Non-dividing cells on this line structure were well aligned, as shown in Figure 9. Three fully spread cells in interphase aligned to the line axes. The cell highlighted with an arrow in the micrograph was undergoing mitosis (possibly in telophase). Figure 9b shows a high magnification micrograph of this dividing cell recorded at a z-plane near the center portion of the cell. The confocal image confirmed that the two emerging daughter cells were near spherical in shape and were not aligned to the line axes.

#### 3.4.5. Isolated Tungsten Lines (1 µm Tungsten Line Inlaid in a SiO_2_ Continuous Phase)

**Figure 10:**
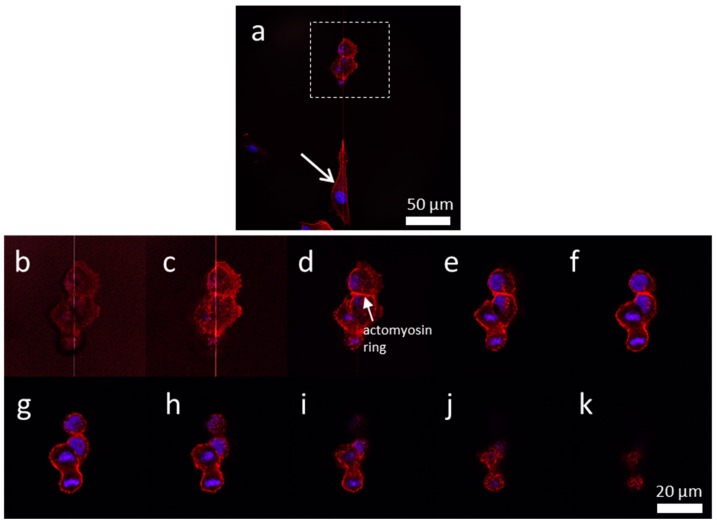
(**a**) Low magnification confocal image of cells adhered to an isolated 1 μm tungsten line. The cell in interphase is labeled with an arrow, while dividing cells are highlighted with a box. Z-stack images (**b**) to (**k**) show the cellular structure near the cell/substrate interface (**b**) and top of the cell (**k**).

One Vero cell appeared to be in the interphase stage of mitosis labelled with an arrow (Figure 10a). This cell was elongated along the tungsten line. Two adjacent cells likely in telophase were on the same isolated line. The cells were compact with a dumbbell shaped geometry. High magnification z-stack images of these dividing cells are shown in Figure 10b,k. The cell at the top appeared to be at the end of the cytokinesis, the final stage of the cell division process, in which the chromosomes appeared decompressed to expanded chromatin. The cell at the bottom appeared to be in telophase, the final stage of mitosis just prior to cytokinesis, showing the condensed chromosomes being pulled to the opposite pole of the cell. Micrographs also showed that the cell division process appeared to follow the Hertwig’s rule where the cell division orientation and the metaphase plate were carried out along the cell long axis [28].

This work proved that manipulating the entire population of cells to align preferentially on engineered biomaterials was hard to achieve. One possible reason was that cells tend to “reset” or alter their geometries during mitosis. Thus, it was unclear how some literature could report that 100% of cells were found to be aligned in the preferred direction of the patterned designs [13,29,30] unless they synchronized the cell cycle for all cells prior to starting their experiments.

## 4. Conclusions

The abilities of the newly developed W-CMP asymmetrical line nanocomposite device to manipulate the alignment behavior of cells were studied. Results demonstrated that the preferential alignment and adhesion behaviors of Vero cells were silicon oxide line width dependent. Moving forward, this will be an important design parameter to consider when designing new tungsten and silicon oxide based nanocomposites. Designing such devices will be easier given that a simple model was shown to predict the propensity of cells to orient themselves based on line width and overall cell size. It should be noted, however, that prior to this study, our hypothesis was that cell division interfered with complete population alignment. Fluorescence confocal micrographs showed that mitotic rounding during cell division reset the cell morphology to an isotropic shape. This provided a possible explanation for why it was difficult to manipulate the entire population of cells to align and elongate in a preferential direction even under the most ideal pattern design and incubation parameters and conditions. Future studies should now be focused on utilizing oriented cells for a specific purpose. In the case of Vero cells, the transfectability of the cells will be further probed. Concomitantly, fully synchronized cultures will be examined to ensure a uniform effect on all cells in the culture.

## Figures and Tables

**Table 1:** Results of adherent cell alignment behaviors on asymmetric patterned comb structures. Tungsten and silicon oxide line widths varied independently in the range of 1 to 100 μm.

Tungsten Line Width (μm)	SiO_2_ Line Width (μm)	Cells Counted (*n*)	Pattern Area (mm^2^)	Density (Cells/mm^2^)	% Cell Distribution ± 10°	% Distribution S.D.
1	1	299	1.7	176	20.7	4.7
1	3	171	1.5	114	32.9	3.2
1	5	164	1.7	96	49.4	2.9
1	9	205	1.8	114	45.3	11.4
1	50	155	1.6	97	60.0	6.4
1	100	135	1.4	96	41.3	9.2
3	1	201	1.6	126	20.4	2.3
5	1	193	1.7	114	28.5	8.1
9	1	169	1.7	99	13.6	5.5
50	1	187	1.6	117	12.3	0.9
100	1	236	1.6	148	9.8	4.4
Blanket W	N/A	282	1.7	166	12.1	0.6

## Supplementary Materials

The following are available online at https://www.mdpi.com/1996-1944/13/2/335/s1: Figure S1: Confocal and electron micrographs of avian cells (QT-35) on the engineered tungsten-silicon oxide nanocomposite. Figure S2: Schematic drawing of the engineered tungsten-silicon oxide surface fabrication processes. Figure S3: Typical scanning electron micrographs of alternating tungsten and silicon oxide lines. Figure S4: Schematic drawing of a cell on the tungsten/silicon oxide patterned comb structure and their orientation parameters. Figure S5: Typical figures used to calculate percent cell area on tungsten and silicon oxide. Figure S6: Z-stack images from near the cell/substrate interface to the top of the cell. Figure S7: Confocal micrographs of adherent cells on blanket tungsten film. Figure S8: A-stack images of a dividing cell possibly in prophase on the structure containing alternating 9 μm tungsten lines and 1 μm silicon oxide lines. Figure S9: Z-stack florescence confocal micrographs of a dividing cell possibly in metaphase and another cell in the interphase stage on the structure containing alternating tungsten and silicon oxide lines of an equal width of 5 μm.
