# Supplementary material for: Limitation in Controlling the Morphology of Mammalian Vero Cells Induced by Cell Division on Asymmetric Tungsten-Silicon Oxide Nanocomposite
Source: Materials (Basel). 2020 Jan 11;13(2):335. doi: 10.3390/ma13020335 (PMC7013836; doi:10.3390/ma13020335)
Supplement: Supplementary file 1 [file materials-13-00335-s001.pdf]

materials

Supporting Information

Limitation in controlling the morphology of mammalian Vero cells induced by cell division on asymmetric tungsten-silicon oxide nanocomposite

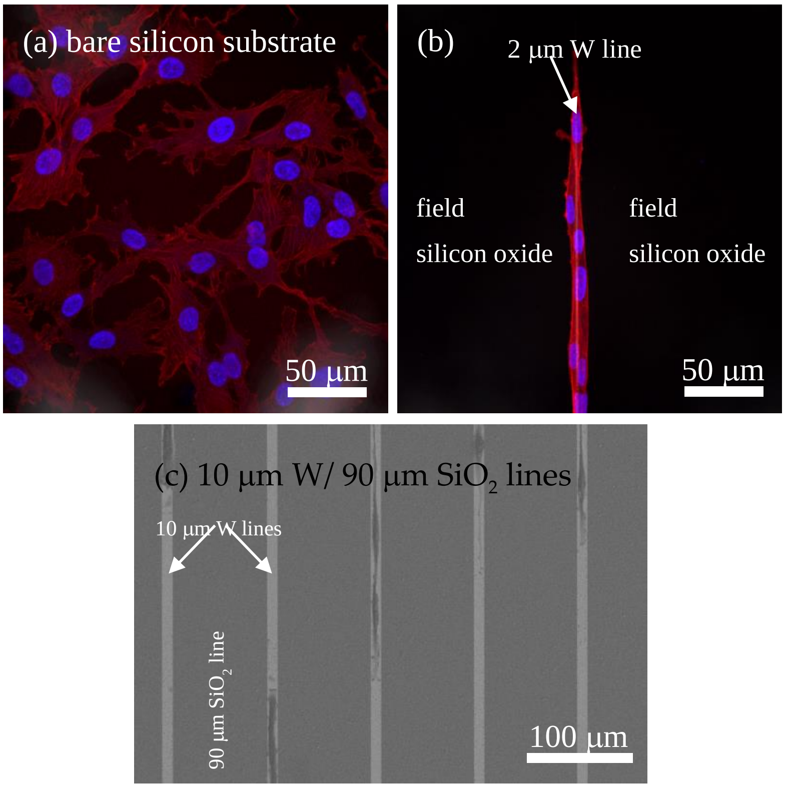

Figure S1(a) show the avian cells evenly spread and randomly oriented on silicon substrate surface without engineering structures. DNA molecules appear blue whereas F-actin microfilaments appear red. In contrast, cells segregated, elongated, and adhered on an isolated tungsten lines with width of 2 µm in a field of silicon oxide (see Figure S1(b)). An electron micrograph of elongated cells adhered on tungsten lines from an alternating tungsten (10 µm) and silicon oxide (90 µm) line pattern is shown in Figure S1(c). These micrographs show QT-35 cells have the same preferential adhesion characteristics as Vero and GM5565 cells described in our prior publications [10, 15].

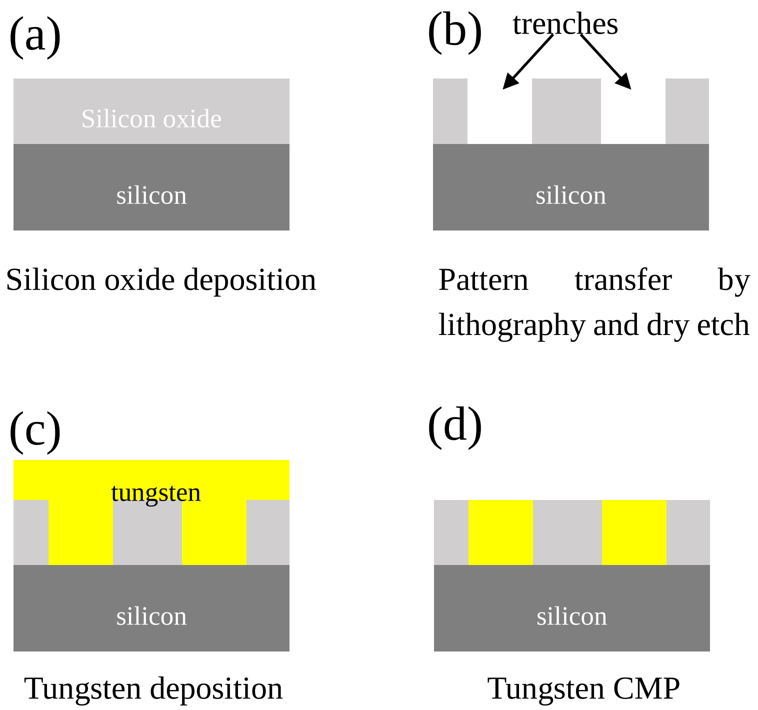

Figure S2. Schematic illustration of the sample preparation process. (a) a thin layer of silicon oxide is deposited on the silicon substrate. (b) Desired patterns are transferred to the substrate by using photolithographic and chemical etch techniques. Tungsten is deposited on the patterned substrate (c). (d) Excess tungsten is removed by using the chemical-mechanical polish technique.

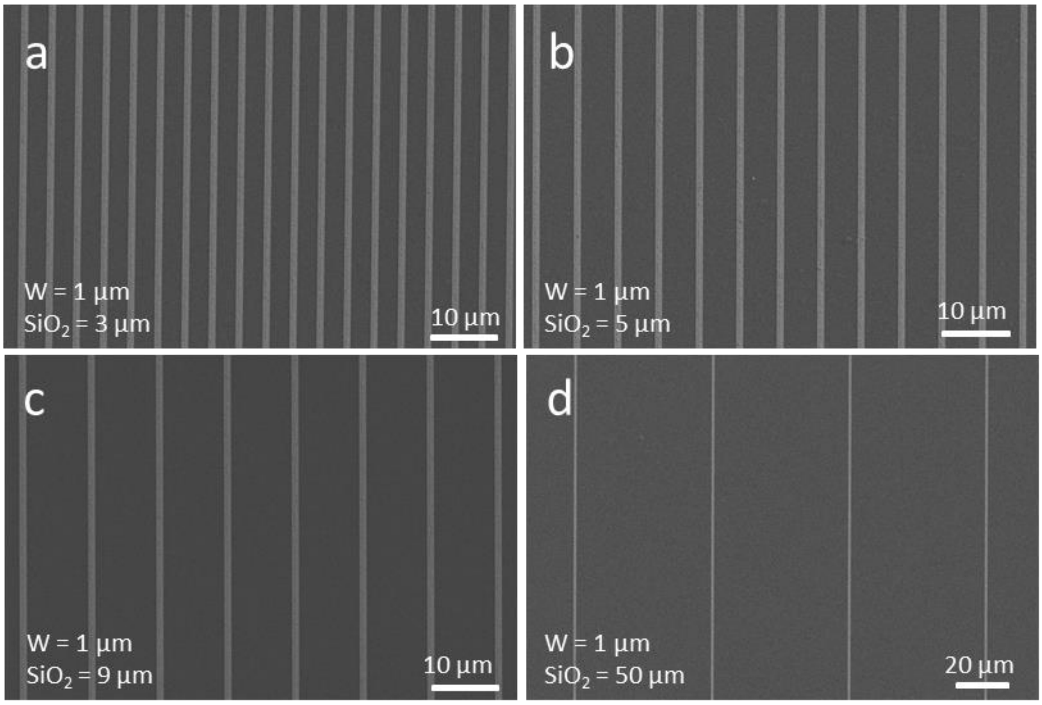

Figure S3. Typical scanning electron micrographs of alternating tungsten and silicon oxide lines with widths of (a) 1 and 3 µm, (b) 1 and 5 µm, (c) 1 and 9 µm, and (d) 1 and 50 µm.

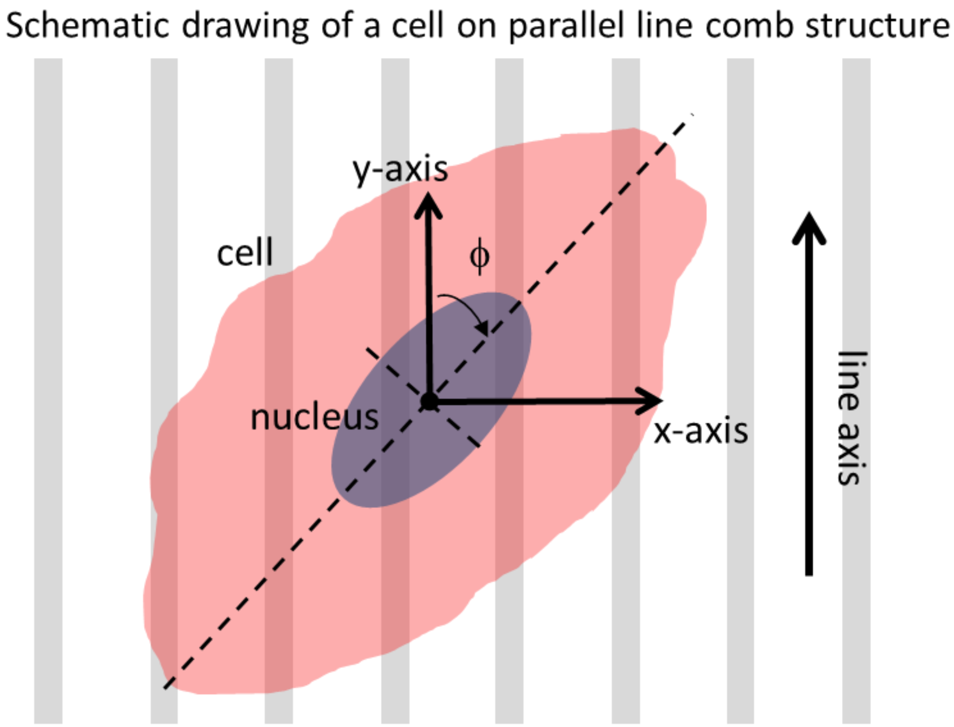

Figure S4. Schematic drawing of a cell on tungsten/silicon oxide patterned comb structure and their orientation parameters.

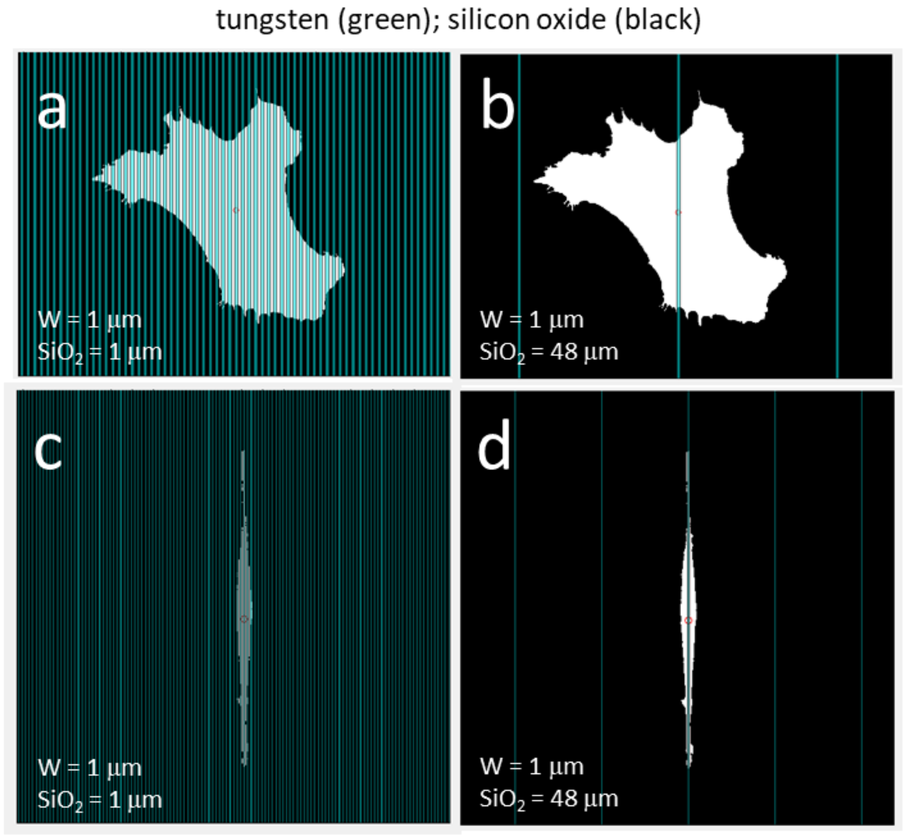

Figure S5. Typical figures used to calculate percent cell area on tungsten (green) and silicon oxide (black). Irregular-shaped and elongated cell models are displayed in (a-b) and (c-d), respectively. (a and c) show modeled comb structures with equal tungsten and silicon oxide line widths of 1 μm. Models of 1 μm wide tungsten lines separated by 48 μm of oxide is shown in (b and d).

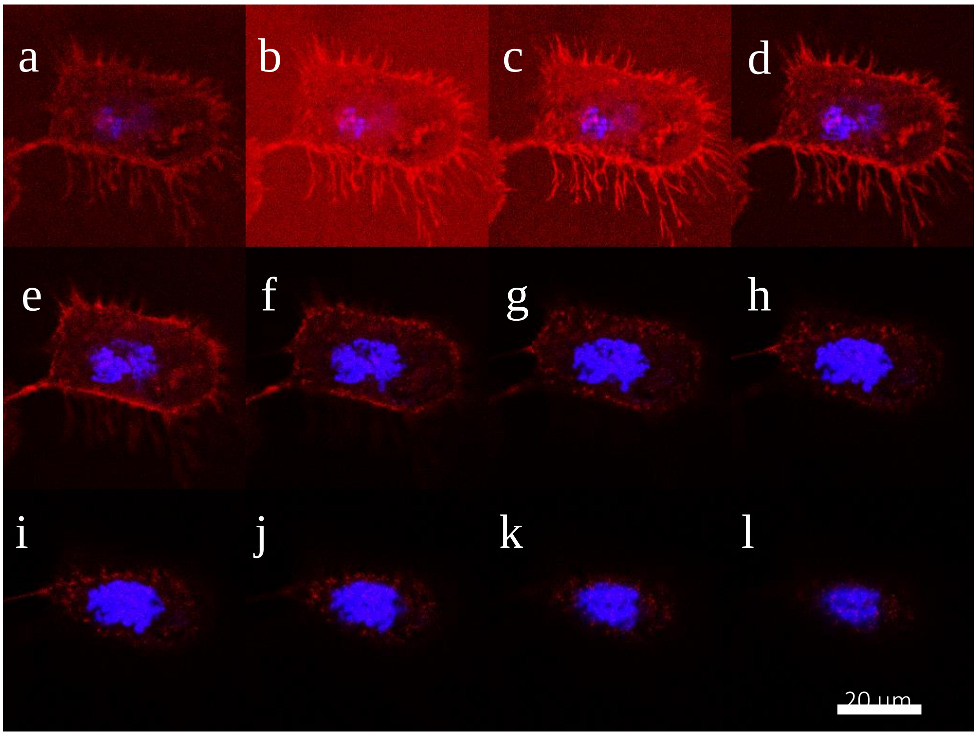

Figure S6. Z-stack images are shown in (a) - (l) from near the cell/substrate interface to the top of the cell. Results show actin filaments protruded from the bottom half of the cell while condensed DNA concentrated near the top of the cell.

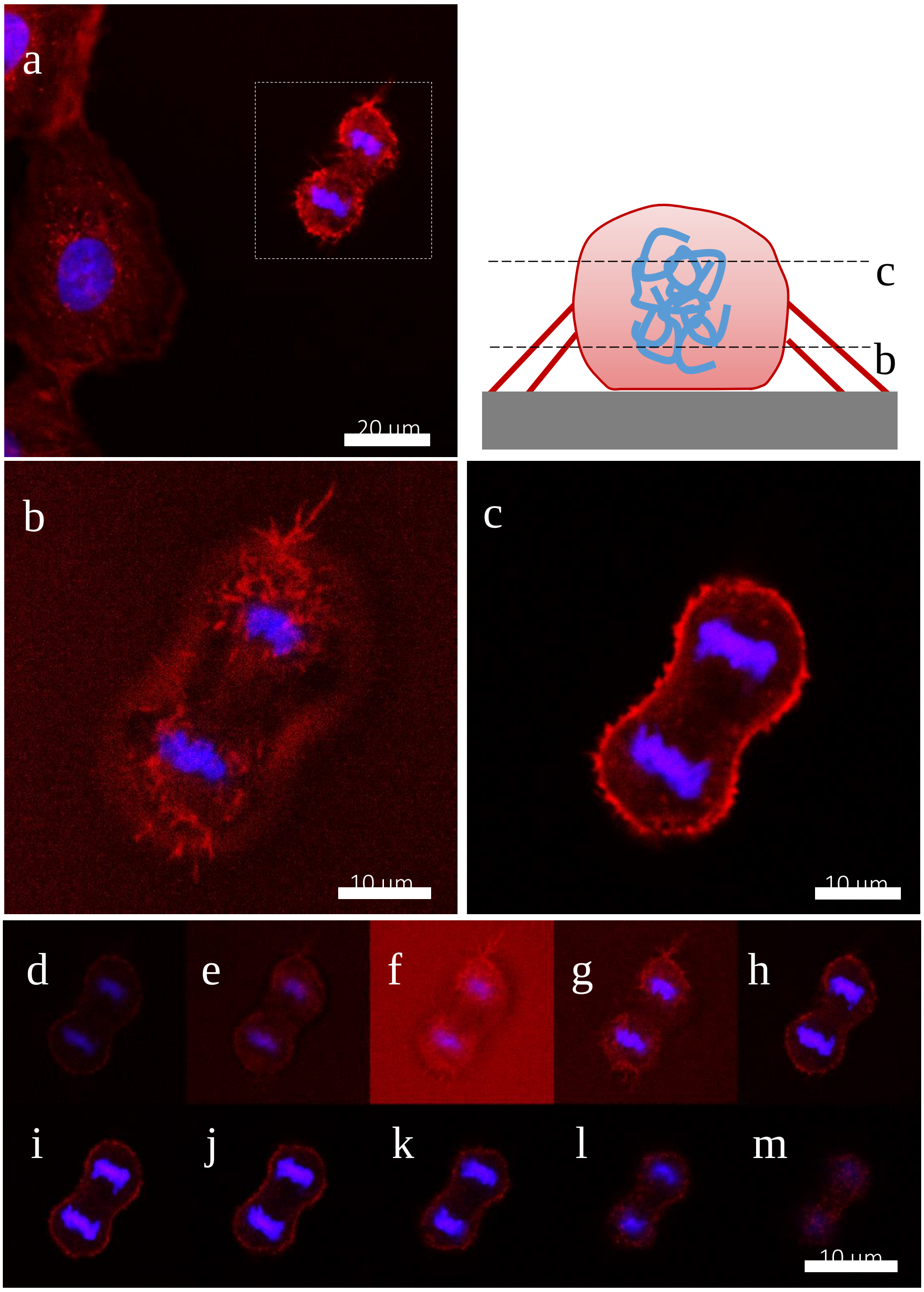

Figure S7. (a) Low magnification confocal micrographs of adherent cells on blanket tungsten film. (b-c) High magnification micrographs of the highlighted cell in (a). (d-m) Z-stack images of cell in (b) from near the interface (d) to the top of cell (m).

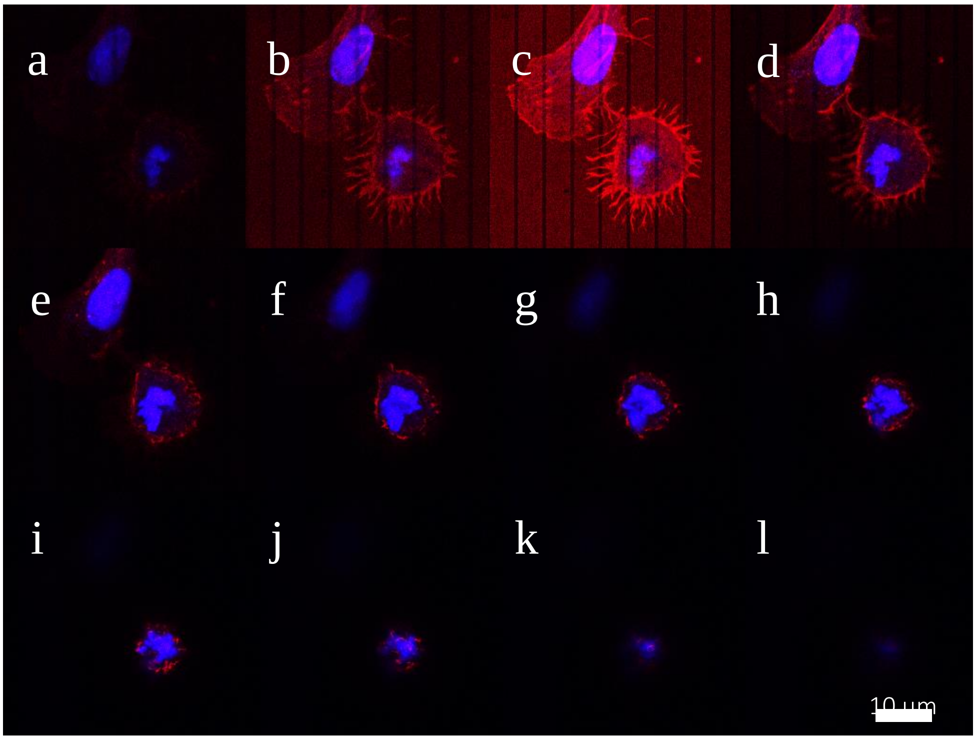

Figure S8. A-stack images (a) – (l) of a dividing cell possibly in the prophase on structure contains alternating 9 μm tungsten line and 1 μm silicon oxide lines. Images were acquired from near the cell/substrate interface (a) to the top of cell (l). It is obvious that the dividing cell is significantly thicker than the cells in interphase stage.

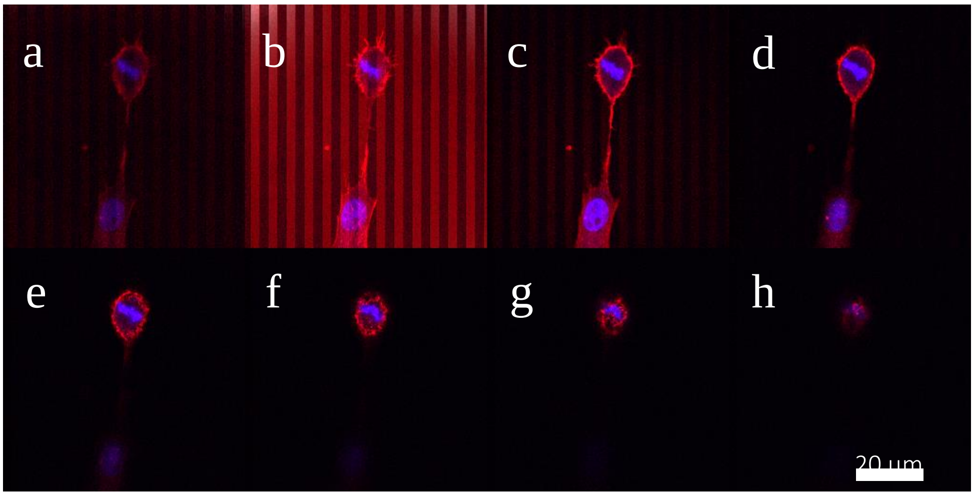

Figure S9. Z-stack florescence confocal micrographs of a dividing cell possibly in the metaphase and another cell in interphase stage on structure contains alternating tungsten and silicon oxide lines of equal width of 5 μm. Images were acquired from near the cell/substrate interface (a) to the top of cell (h).

© 2020 by the authors. Submitted for possible open access publication under the terms and conditions of the Creative Commons Attribution (CC BY) license (http://creativecommons.org/licenses/by/4.0/).
